# Determining the Optimal Energy Level of Virtual Monoenergetic Images in Dual-Source CT for Diagnosis of Bowel Obstruction and Colitis

**DOI:** 10.3390/diagnostics13233491

**Published:** 2023-11-21

**Authors:** Loris Lahuna, Joël Greffier, Jean Goupil, Julien Frandon, Maxime Pastor, Fabien De Oliveira, Jean Paul Beregi, Djamel Dabli

**Affiliations:** Department of Medical Imaging, IMAGINE UR UM 103, Montpellier University, Nîmes University Hospital, Bd Prof Robert Debré, CEDEX 9, 30029 Nîmes, Francejean.goupil@chu-nimes.fr (J.G.); jean.paul.beregi@chu-nimes.fr (J.P.B.)

**Keywords:** dual-energy CT, dual-source CT, colitis, bowel obstruction, virtual monoenergetic images

## Abstract

Images from 64 patients undergoing an enhanced abdominal-pelvis scan at portal phase in dual-energy CT mode for the diagnosis of colitis or bowel obstruction were retrospectively analyzed. Acquisitions were performed on a third-generation dual-source CT (DSCT) 100/Sn150 kVp. Mixed images were generated, as well as virtual monoenergetic images (VMIs) at 40/50/60/70 keV. Objective image quality was assessed on VMIs and mixed images by measuring contrast, noise and contrast-to-noise ratio (CNR). Noise, smoothing and overall image quality were subjectively analyzed by two radiologists using Likert scales. For both patient groups, the noise decreased significantly according to the energy level from 40 to 60 keV by −47.2 ± 24.0% for bowel obstruction and −50.4 ± 18.2% for colitis. It was similar between 60 and 70 keV (*p* = 0.475 and 0.059, respectively). Noise values were significantly higher in VMIs than in mixed images, except for 70 keV (*p* = 0.53 and 0.071, respectively). Similar results were observed for contrast values, with a decrease between 40 and 70 keV of −56.3 ± 7.9% for bowel obstruction −56.2 ± 10.9% for colitis. The maximum CNR value was found at 60 keV compared to other energy levels and mixed images, but there was no significant difference with the other energy levels apart from 70 keV (−9.7 ± 9.8%) for bowel obstruction and 40 keV (−6.6 ± 8.2%) and 70 keV (−5.8 ± 9.2%) for colitis. The VMIs at 60 keV presented higher scores for all criteria for bowel obstruction and colitis, with no significant difference in smoothing score compared to mixed images (*p* = 0.119 and *p* = 0.888, respectively).

## 1. Introduction

In recent years, dual energy computed tomography (DECT) has seen its applications and use for abdomen-pelvic CT examinations grow [1]. This is mainly due to the variety of images produced, improving the detection and characterization of abdominal lesions such as renal, hepatic, pancreatic and acute abdominal lesions [1,2,3,4,5,6]. This technique is based on the use of two X-ray spectra: one at low energy, the other at high energy. These spectra are used by material decomposition algorithms to characterize tissues according to two or three basic materials, including those with a low atomic number (e.g., water) and those with a high atomic number (e.g., iodine) [7,8].

In third-generation dual source CT (DSCT), the two spectra are obtained with two X-ray tube/detector pairs (95° out of phase). Low (70 to 100 kVp) and high kVp images are generated (140 kVp and Sn150 kVp) [9] and used to produce mixed images simulating single energy CT (SECT) images similar to those produced at 120 kVp with higher photon statistics than low- and high-kVp images [10]. In addition, material-specific images, such as iodine maps, are calculated to quantify iodine concentration in lesions, and virtual noncontrast (VNC) images can be generated to obtain images, virtually, without iodine [11,12,13,14]. Finally, virtual monochromatic images (VMIs) are also produced, which are useful for improving iodine contrast, using low energy levels and reducing the image artifacts found with high energy levels [15,16,17,18,19]. These different images generated by DECT scanners have improved the diagnosis of various pathologies, such as the discrimination of intraperitoneal hematoma and intestinal structures [20], or the improvement of stent visualization [21].

Several phantom studies have demonstrated a higher contrast-to-noise ratio (CNR) and detectability on VMIs for the lowest energy levels, ranging from 40 to 70 keV, compared to SECT images [22,23]. These studies have shown that the iodine contrast in VMIs increases as the energy level increases, and the opposite is found for image noise. Since detectability is affected by noise and contrast, any parameters influencing them will have an impact on detectability. These parameters include the performance of the DECT platform, the anatomical region and iodine uptake of the lesion, which can all affect image noise and contrast [24,25,26,27,28,29,30]. Other studies on patients have reported similar results concerning noise, contrast and CNR, and highlighted the interest of the low energy levels of VMIs to improve the diagnosis of various abdominal pathologies [31,32,33,34,35,36,37]. These studies report different optimal energy levels ranging from 40 to 70 keV to be used according to the clinical indication and DECT platform. However, most of these studies focus on the detectability and characterization of solid organ lesions, in particular hepatic, renal or pancreatic lesions. Indeed, few studies have specifically studied the contribution of VMIs in the context of emergency pathologies such as bowel obstruction [38] and colitis, or defining the right energy level to use for the various types of clinical indication [39,40,41,42,43]. These levels have either been determined for pathologies other than bowel obstruction and colitis, or for an older generation DSCT or systems other than DSCT platforms.

The purpose of this study was to compare the objective and subjective image quality of the four lowest monoenergetic levels of VMIs and mixed images obtained with third-generation DSCT, in order to determine the most suitable energy level for diagnosing bowel obstruction and colitis in an emergency context. 

## 2. Materials and Methods

### 2.1. Design and Study Population

This retrospective monocentric study was approved by our institutional review board (23.03.06). A letter was sent to all patients informing them of the use of their images in this study and their right to oppose.

Reports of CT scans performed in emergency departments from February 2019 to February 2020 with portal-phase DECT acquisition of the abdomen-pelvis were screened from the Picture Archiving and Communication System (PACS). 

**Figure 1:**
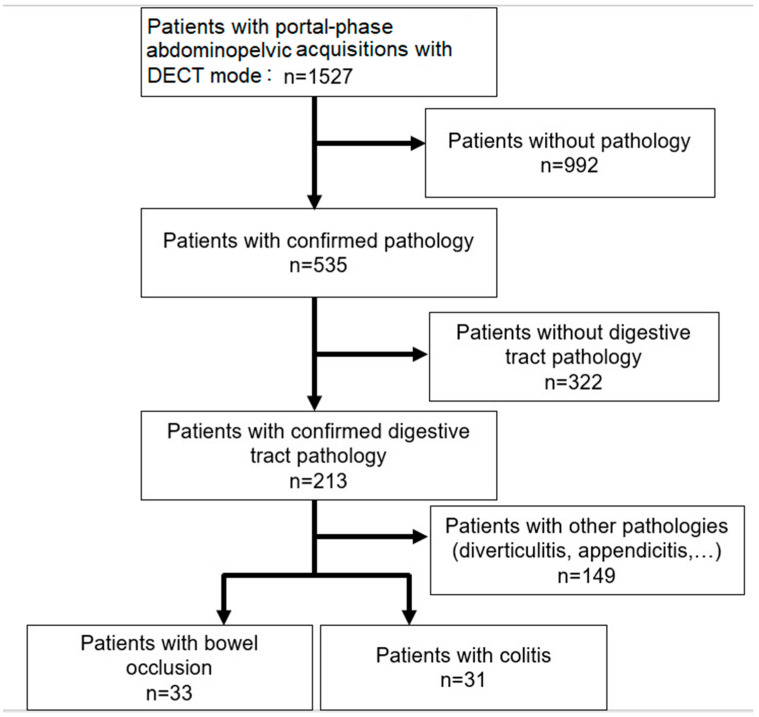
Flowchart showing patient inclusion for the two patient groups.

Over the study period, all patients with a digestive tract pathology were initially selected. Then, only patients with confirmed digestive pathology in the examination report were considered. Finally, among the confirmed digestive pathologies, only those for which we had at least 30 patients were included, in order to have a statistically significant sample for each pathology. Then, two pathologies were included in the study: intestinal obstruction and colitis. Figure 1 shows the flowchart for the selection of patients included. The final patient population was divided into two groups according to pathology: one for bowel obstruction and one for colitis.

### 2.2. Acquisition and Reconstruction Parameters

Acquisitions were performed on a Somatom Force DSCT (Siemens Healthineers, Forchheim, Germany) using the clinical routine protocol for an abdominal pelvis examination used and validated by the department′s radiologists for the past 2 years. Acquisitions were performed at the portal phase by injecting 1 cm^3^ of Xenetix 300 contrast medium per 1 kg of patient weight. The acquisition parameters were: a kVp pair of 100/Sn150 kVp, a rotation time of 0.5 s/rot, a pitch factor of 0.6 and a beam collimation of 128 × 0.6 mm. The automatic current modulation system (CareDose 4D, Siemens Healthineers, Forchheim, Germany) was enabled with the reference quality tube current set at 190 mAs for 100 kVp and 95 mAs for Sn150 kVp. 

Raw data were reconstructed using a soft kernel (Br40), a 1 mm slice thickness (1 mm overlapped) and Level 3 of the Advanced Modeled Iterative Reconstruction (ADMIRE) algorithm. For each patient, mixed images were generated using a dual energy composition parameter of 0.8 [10], as well as VMIs at 4 energy levels: 40, 50, 60 and 70 keV.

### 2.3. Dosimetric Analysis

Volume CT dose index (CTDI_vol_) and dose length product (DLP) values of the DECT acquisition of the abdomen-pelvic were extracted from the dose archiving and communication system (DACS) for all patients included. To compensate for the lack of data on patients’ weight and height, a dose was estimated, taking into account the morphology of the patients, using the size-specific dose estimates (SSDE) according to the methodology of the American Association of Physicists in Medicine Task Group 204 [44]. 

The mean values of CTDI_vol_, DLP and SSDE were calculated and compared between the two patient groups. 

### 2.4. Objective Image Quality Assessment

For all images, one region of interest (ROI) of 1 cm² was placed by an experienced radiologists (10 years of experience) on the pathological area of the bowel and one similar ROI on the subcutaneous fat on the SyngoVia software (version VBA60A_HF03, Siemens Healthineers). The mean Hounsfield unit (HU) value of each ROI (〖HU〗_(bowel area) and 〖HU〗_fat) was calculated and the standard deviation of HU representing the image noise was calculated for the ROI placed on fat (〖SD〗_fat). The contrast value was calculated by the difference between 〖HU〗_(bowel area) and 〖HU〗_fat. The contrast-to-noise ratio (CNR) was calculated for each image using the following formula:

CNR = (〖HU〗_(bowel area)-〖HU〗_fat)/〖SD〗_fat(1)

### 2.5. Subjective Image Quality Assessment

The quality of mixed images and VMIs at the 4 single-energy levels was evaluated by two radiologists, one experienced (10 years of experience) and one junior (3 years of experience). Both radiologists were blinded to the type of images during the evaluation. 

Images were scored on 3 image quality criteria: noise, image smoothing and overall image quality, according to the following scale: 1 = unacceptable, 2 = suboptimal, 3 = acceptable, 4 = above average, 5 = excellent. 

Images with a score of less than 3 on the overall quality criterion were considered as insufficient for clinical use. 

### 2.6. Statistical Analysis

Statistical analyses were performed for each patient group using GMRC Shiny Stats software version 2.0 [45]. Overall agreement between the two radiologists’ scores was calculated with Cohen′s Kappa test and classified as poor (κ = 0.00–0.20), low (κ = 0.21–0.40), moderate (κ = 0.41–0.60), good (κ = 0.61–0.80) or excellent (κ = 0.81–1.00). The Mann–Whitney U test for impaired samples was used to compare dosimetric data between the two patient groups. Finally, the Wilcoxon test for paired samples was used for the mean CNR and the mean scores of each criterion for the two radiologists’ comparisons between each image type and each patient group.

## 3. Results

### 3.1. Study Population

The total number of patients included was 64, with 33 for the bowel obstruction group and 31 in the colitis group (Figure 1). The mean age of patients was 65 ± 20 years (range: 18 to 95) and 49 ± 22 (range: 20 to 92), respectively. There were 16 female patients in the bowel occlusion group and 17 in the colitis group. The symptoms justifying the CT examination were mostly occlusive syndrome (*n* = 25) for the bowel obstruction group and pain (*n* = 23) for colitis.

### 3.2. Dosimetric Analysis

Dosimetric data and associated *p* values are depicted in Table 1. The mean CTDI_vol_ for bowel obstruction was significantly higher than for colitis by 34.2 ± 1.5% (range: −1.3% to 127.8). Similar results were observed for DLP by 40.4 ± 1.3% (range: −7.8 to 101.8), and effective diameter (deff) by 18.9 ± 5.1% (range: 9 to 34). No significant difference was found for SSDE. The difference between the CTDI_vol_ and SSDE was lower for patients with bowel obstruction than for those with colitis.

### 3.3. Objective Image Quality Assessment

**Figure 2:**
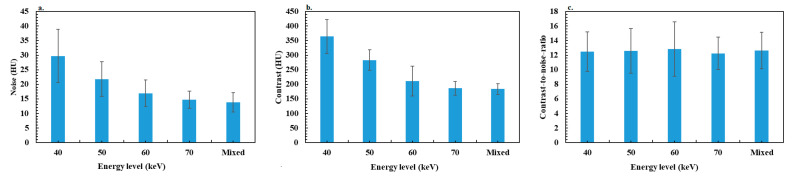
Results of objective image quality assessment for the bowel obstruction group: (**a**) noise, (**b**) contrast and (**c**) contrast-to-noise ratio.

**Figure 3:**
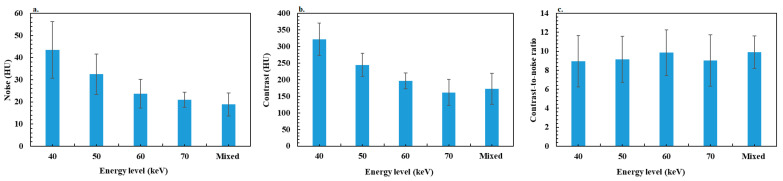
Results of objective image quality assessment for the colitis group: (**a**) noise, (**b**) contrast and (**c**) contrast-to-noise ratio.

Figure 2 shows the variation in noise, contrast and CNR according to the energy levels of VMIs and for mixed images for bowel obstruction. Figure 3 shows the results for colitis. The *p* values resulting in data comparisons of all objective image quality metrics are depicted in Table 2 for bowel occlusion and Table 3 for colitis. 

For patients with bowel obstruction, the noise decreased significantly according to the energy level from 40 to 60 keV by −47.2 ± 24.0% (range: −20.8 to −75.6), and was similar between 60 and 70 keV. The noise values were significantly higher for VMIs than for mixed images, except for 70 keV, 6.5 ± 6.1% (range: −16.7–8.3%). Similar results were observed for contrast values with a decrease between 40 and 70 keV of −56.3 ± 7.9% (range: −35.2 to −68.2), but all differences between image types were significant except between mixed images and VMIs at 70 keV with a difference of 0.9 ± 9.1% (range: −16.9 to −20.9). Similar outcomes were found for colitis, with a significant decrease in noise and contrast between 40 and 60 keV of −50.4 ± 18.2% (range: −4.8 to −76.3) and −56.2 ± 10.9% (range: −17.8 to −65.9), respectively. 

For both patient groups, the maximum CNR value was found at 60 keV compared to other energy levels and mixed images. However, no significant difference was observed with mixed images and with other energy levels, except with 70 keV (−9.7 ± 9.8% (range: 5.9 to −30.7)) for bowel obstruction and 40 keV (−6.6 ± 8.2% (range: 4.8 to −21.4)) and 70 keV (−5.8 ± 9.2% (range: 6.3 to −32.1)) for colitis.

### 3.4. Subjective Image Quality Assessment

The results showed good interobserver agreement for the analysis of the different criteria with a calculated kappa index of 0.95 for noise, 0.89 for smoothing, 0.88 for overall quality, 0.91 for diagnostic quality and 0.99 for diagnostic confidence. The *p* values resulting in data comparisons of all subjective image quality criterion are depicted in Table 4 for bowel occlusion and Table 5 for colitis. 

**Figure 4:**
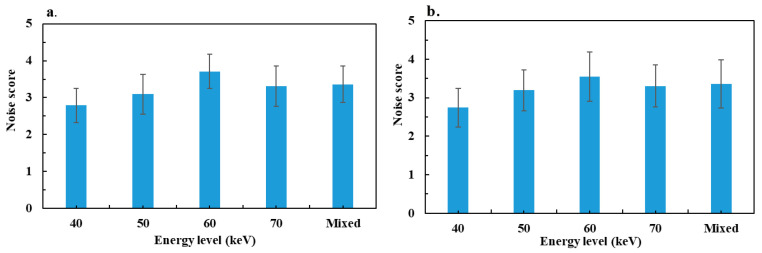
Results of mean noise scores assessed by two radiologists: (**a**) bowel obstruction group and (**b**) colitis group.

The variations in mean noise scores for both pathologies are depicted in Figure 4. For patients with bowel obstruction, the maximum noise score was found in VMIs at an energy level of 60 keV. It increased significantly between 40 and 60 keV, and then decreased significantly between 60 and 70 keV. The score for mixed images was significantly higher than the scores for energy levels of 40 and 50 keV, significantly lower than with 60 keV and similar with an energy level of 70 keV. For colitis, similar results were obtained with the maximum score at 60 keV, and no significant difference between mixed images and the 70 keV score.

**Figure 5:**
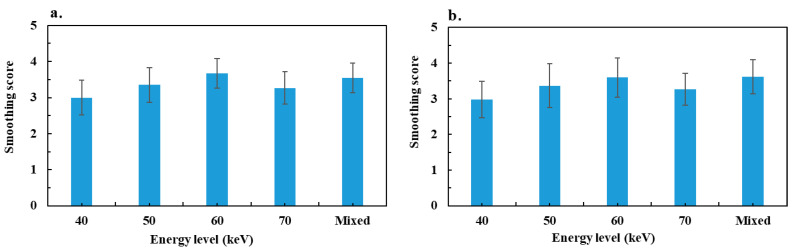
Results of mean image smoothing score assessed by two radiologists: (**a**) bowel obstruction group and (**b**) colitis group.

Figure 5 shows the image smoothing score for the two groups. For bowel obstruction, the maximum smoothing score was reported for VMIs at 60 keV. It increased significantly between 40 and 60 keV, and decreased significantly at 70 keV. The mixed image score was significantly higher than for images at 40, 50 and 70 keV, but not significant for 60 keV. For colitis, similar results were observed with the maximum score at 60 keV, and no significant difference between mixed images and the 60 keV score.

**Figure 6:**
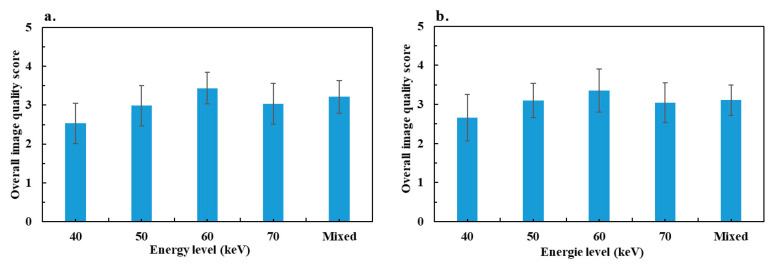
Results of the mean overall image quality score assessed by two radiologists: (**a**) bowel obstruction group and (**b**) colitis group.

Figure 6 shows the overall image quality scores according to the energy levels and mixed images. For the two groups, the maximum score observed was at 60 keV. It increased significantly from 40 to 60 keV, and decreased significantly at 70 keV. The mixed image score was significantly lower than the score at 60 keV and significantly higher that at 40 and 50 keV, except for colitis, where the difference was not significant at 50 keV. The difference between mixed images and VMIs at 70 keV was not significant for either group. Finally, all VMIs at 60 and 70 keV, as well as mixed images, had a score ≥ 3, but some of the VMIs at 40 and 50 keV were of insufficient diagnostic quality (24% of patients at 40 keV and 12% at 50 keV for occlusions, and 13% and 3%, respectively, for colitis).

**Figure 7:**
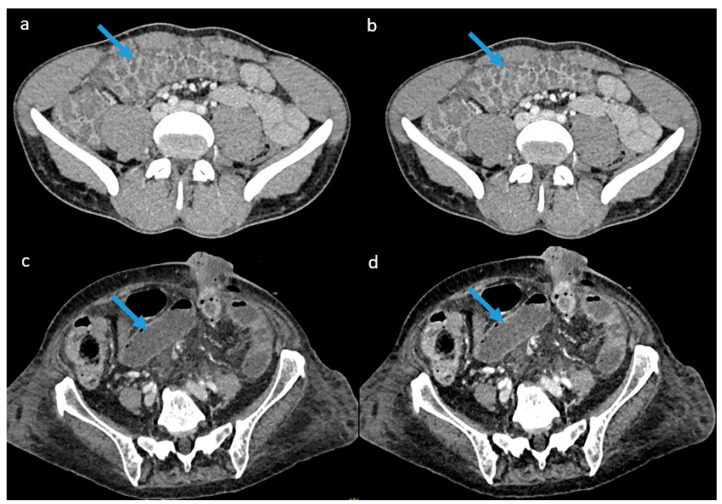
Example of images obtained for both pathologies: (**a**) mixed image generated for a 39-year-old male patient with colitis, (**b**) virtual monoenergetic image at 60 keV for the same patient. (**c**) mixed image generated for an 81-year-old female patient with bowel obstruction, and (**d**) virtual monoenergetic image at 60 keV for the same patient. Blue arrows indicate the pathological area.

Figure 7 below shows examples of mixed images and VMIs at 60 keV for two cases, one of colitis and one of bowel obstruction. 

## 4. Discussion

In this study, the image quality of VMIs at 40, 50, 60 and 70 keV, as well as mixed images generated by the DSCT platform, for the diagnosis of bowel obstruction and colitis in the emergency department were compared objectively and subjectively. The results showed that the maximum CNR was observed on VMIs at 60 keV compared to other VMIs and mixed images, but without statistical significance. The subjective evaluation showed that the VMIs at 60 keV had the best score values for noise, smoothing and overall quality with a significant difference compared to the other energy levels except for smoothing between 60 keV and mixed images, which were considered similar for both pathologies. 

The mean CTDI_vol_ for patients with bowel obstruction was significantly higher than that of patients with colitis. This can be explained mainly by the patients’ corpulence. Indeed, in the absence of data on the patients’ weight and height, the calculation of the deff showed that patients with bowel obstruction had a significantly higher diameter than those with colitis. Using the automatic current modulation system therefore increased the mAs for these patients in order to maintain sufficient image quality. This is also confirmed by the difference observed between the SSDE and CTDI_vol_, which was lower for patients with bowel obstruction than with colitis because a deff was lower than the reference diameter of the standard dosimetry phantom (32 cm) in the case of the bowel occlusion group, and similar for the colitis group. In addition, the CTDI_vol_ results showed that the average CTDI_vol_ and DLP of the acquisitions were lower than the national diagnostic reference levels, which are 13 mGy for CTDI_vol_ and 625 mGy × cm for DLP. This reflects the optimized practices and protocols for dual-energy CT acquisition at our institution.

Concerning the objective image quality assessment, a decrease in iodine contrast and noise was observed as the energy level increased in VMIs. This was also reported in several studies [19,22,23], and may be explained by the strong attenuation of low-keV X-rays due to the increased contribution of the photoelectric effect. It results in an increase both in the contrast between the iodine and the surrounding tissue at low keV and noise. This can be explained by the fact that the 70 keV energy level is close to the average energy of the X-ray spectrum of a conventional SECT acquisition at 120 kVp, represented by the mixed images in this case. Nevertheless, the comparison of the CNR values showed a trend toward a maximum value at 60 keV, but the difference was significant only compared to 70 keV for bowel obstruction and 40 keV, 70 keV and mixed images for colitis. Indeed, the decrease in contrast between 40 and 60 keV is compensated by a more significant decrease in noise, which improves CNR. This compensation effect is less marked between 60 and 70 keV and with mixed images, which explains the drop in CNR. These results are different from those reported on phantom studies showing that CNR peaked at 40 keV and decreased as the energy levels decreased [22,46].

The results of the subjective image quality assessment showed that the noise score increased between 40 and 60 keV and then decreased at 70 keV. The score for those levels was similar to that of the mixed images. These results are consistent with the objective noise assessment except between 60 and 70 keV in both groups, where the difference in the objective noise between 60 and 70 keV was not significant. Radiologists have a better perception of images at 60 keV for noise. For image smoothing, the radiologists evaluated the best noise texture on the 60 keV images for VMIs, for both pathologies, and no significant difference was observed between these images and the mixed images. This result is similar to those reported by Greffier et al. [22], who found an increase in the average frequency of the noise spectrum between 40 and 60 keV, and then a decrease at 70 keV, reflecting a lesser smoothing effect at 60 keV. The best overall image quality was observed on the VMIs at 60 keV for the two pathologies. This score reflects the best compromise between noise and smoothing for visualizing bowel obstruction and colitis. A different result was observed in a phantom study using a task-based image quality assessment, including detectability index, on the same DECT platform [22]. That study showed that detectability was maximal at 70 keV for lesions simulated with blood and iodine contrast at 2 mg/mL. The difference observed between that phantom study and the results of our study can be explained by a greater iodine contrast in bowel occlusion and colitis compared to the simulated contrast in the study. 

Some of the VMIs at 40 and 50 keV were of insufficient quality for clinical use (24% of patients at 40 keV and 12% at 50 keV for bowel occlusion, and 13% and 3%, respectively, for colitis). The trade-off between noise and smoothing associated with image aspects, clear visualization of edges and structures of diagnostic interest thus justified the better quality on VMIs at 60 keV compared to other monoenergetic levels and mixed images. 

Finally, the results presented in this study confirmed the interest of low keV VMIs compared to conventional images at 120 kVp. The results suggest that the most suitable energy level for the diagnosis of bowel occlusion and colitis is 60 keV for examinations performed on the third-generation DSCT scanner. These results are different from those of Darras et al. [40], who found that the 70 keV level was the most suitable for the diagnosis of small bowel occlusion for examinations performed on second-generation DSCT. This difference could therefore be explained mainly by the difference in generation of the DSCT scanner used. Indeed, the improvement in spectral performance of third-generation DSCT compared to second-generation DSCT, particularly with the combination of a higher voltage and the use of a tin filter on the second tube [47], led to greater image quality at lower energy levels on VMIs. Another experimental study, performed on pigs by Potretzke TA et al. [41], reported a significant improvement in conspicuity of the ischemic bowel on VMIs at 51 keV compared with SECT. Yet, this study was performed on pigs, not patient data, and involved a kV-switching CT which could explain the difference from our results. The 40 keV energy level was also reported by Lee et al. [42] for bowel wall change in Crohn’s disease, but using a dual-layer CT scanner. Finally, Zins et al. [43] reported examples of improved image quality compared to SECT for the diagnosis of small bowel obstruction with VMIs at 65 keV, which is closer to our results.

This study has certain limitations, particularly its monocentric nature, making it difficult to generalize the results to all other centers. Additionally, the analysis was performed retrospectively, and it only concerned a single CT model. Finally, the lack of patients’ weight and height data is also a limitation, although this was compensated for by calculating the SSDE. 

## 5. Conclusions

This study shows the interest of using VMIs to evaluate bowel obstruction and colitis in an emergency setting. Using VMIs at an energy level of 60 keV may allow a better image quality in terms of noise, image texture, and overall quality. 

## Figures and Tables

**Table 1:** Dosimetric data for both examination types and *p* value corresponding to the comparison between both groups.

		Colitis	Bowel Occlusion	*p* Value
Dosimetric data	DLP (mGy × cm)	366.13 ± 108.53 (198.50–630.75)	514.05 ± 245.62 (183.06–1272.6)	0.004 *
	CTDI_vol_ (mGy)	7.45 ± 1.84 (4.53–12.04)	10.00 ± 4.60 (4.08–27.43)	0.005 *
	Effective diameter (cm)	26.77 ± 4.00 (18.71–35.50)	31.83 ± 3.77 (25.07–38.68)	<0.001 *
	SSDE (mGy)	10.25 ± 2.03 (6.26–18.42)	11.23 ± 4.31 (4.31–26.06)	0.852

Values are expressed as mean ± standard deviation (min–max). * Indicates significant difference.

**Table 2:** *p* values issued from the comparison of noise, contrast and contrast-to noise ratio between four energy levels of VMIs and mixed images for the bowel occlusion group.

Image Quality Metric	Energy Levels (keV)	Mixed Image	70	60	50
Noise	40	<0.001 *	<0.001 *	<0.001 *	<0.001 *
	50	<0.001 *	<0.001 *	<0.001 *	-
	60	<0.001 *	0.475	-	-
	70	0.530	-	-	-
Contrast	40	<0.001 *	<0.001 *	<0.001 *	< 0.001 *
	50	<0.001 *	<0.001 *	<0.001 *	-
	60	<0.001 *	<0.001 *	-	-
	70	0.360	-	-	-
CNR **	40	0.314	0.608	0.262	0.664
	50	0.977	0.294	0.903	-
	60	0.315	0.041	-	-
	70	0.245	-	-	-

* Indicates significant difference. ** Contrast-to-noise ration.

**Table 3:** *p* values issued from the comparison of noise, contrast and contrast-to noise ratio between four energy levels of VMIs and mixed images for the colitis group.

Image Quality Metric	Energy Levels (keV)	Mixed Image	70	60	50
Noise	40	<0.001 *	<0.001 *	<0.001 *	0.006 *
	50	<0.001 *	<0.001 *	<0.001 *	-
	60	<0.001 *	0.059	-	-
	70	0.071	-	-	-
Contrast	40	<0.001 *	<0.001 *	<0.001 *	<0.001 *
	50	<0.001 *	<0.001 *	<0.001 *	-
	60	<0.001 *	0.001 *	-	-
	70	0.135	-	-	-
CNR **	40	0.083	0.484	0.048	0.857
	50	0.064	0.595	0.095	-
	60	0.294	0.045	-	-
	70	0.066	-	-	-

*: Indicates significant difference. ** Contrast-to-noise ration.

**Table 4:** *p* values issued from the comparison of subjective image quality criterion: noise, smoothing and overall image quality for four energy levels of VMIs and mixed images for the bowel occlusion group.

Image Quality Criterion	Energy Levels (keV)	Mixed Image	70	60	50
Noise	40	<0.001 *	<0.001 *	<0.001 *	<0.001 *
	50	<0.001 *	0.039 *	<0.001 *	-
	60	0.001 *	0.010 *	-	-
	70	0.521	-	-	-
Smoothing	40	<0.001 *	<0.001 *	<0.001 *	<0.001 *
	50	0.035 *	0.513	<0.001 *	-
	60	0.119	<0.001 *	-	-
	70	0.005 *	-	-	-
Overall image quality	40	<0.001 *	<0.001 *	<0.001 *	<0.001 *
	50	0.023 *	0.476	<0.001 *	-
	60	0.009 *	0.001 *	-	-
	70	0.283	-	-	-

*: Indicates significant difference.

**Table 5:** *p* values issued from the comparison of subjective image quality criterion: noise, smoothing and overall image quality for four energy levels of VMIs and mixed images for the colitis group.

Image Quality Criterion	Energy Levels (keV)	Mixed Image	70	60	50
Noise	40	<0.001 *	<0.001 *	<0.001 *	<0.001 *
	50	0.014 *	0.893	<0.001 *	-
	60	0.029 *	0.004 *	-	-
	70	0.066	-	-	-
Smoothing	40	<0.001 *	<0.001 *	<0.001 *	<0.001 *
	50	0.014 *	0.976	<0.001 *	-
	60	0.888	0.016 *	-	-
	70	0.030 *	-	-	-
Global image quality	40	<0.001 *	<0.001 *	<0.001 *	<0.001 *
	50	0.162	0.311	<0.001 *	-
	60	0.043 *	<0.001 *	-	-
	70	0.057	-	-	-

*: Indicates significant difference.

## Data Availability

Data available upon request from the corresponding author.
